# Lenvatinib-induced pemphigus erythematosus in hepatocellular carcinoma: a unique case report

**DOI:** 10.3389/fonc.2025.1505596

**Published:** 2025-03-25

**Authors:** Xiaoqing Li, Suhua Ma, Qing She, Zirong Liu, Yanan Liu, Yanjing Kuang, Xiaozhun Huang, Zhengyin Zhan

**Affiliations:** Department of Hepatobiliary Surgery, National Cancer Center/National Clinical Research Center for Cancer/Cancer Hospital & Shenzhen Hospital, Chinese Academy of Medical Sciences and Peking Union Medical College, Shenzhen, China

**Keywords:** lenvatinib, hepatocellular carcinoma, pemphigus, adverse drug reaction, case report

## Abstract

Adjuvant lenvatinib in combination with transarterial chemoembolization (TACE) has demonstrated prolonged disease-free survival in hepatocellular carcinoma patients at high risk of recurrence post-resection. Here, we present the case of a 68-year-old woman who developed serious side effects including pemphigus erythematosus (PE) linked to lenvatinib usage. Initially treated for breast cancer with radical surgery in April 2018 followed by adjuvant therapy, she was later diagnosed with liver cancer, initially mistaken for metastatic breast cancer to the liver. Although systemic treatment for advanced breast cancer was received, the tumor continued to progress and required partial removal of the liver after final evaluation. Subsequent pathology revealed hepatocellular carcinoma combined with risk factors for recurrence, prompting adjuvant therapy with TACE and oral lenvatinib. After three weeks of lenvatinib administration, the patient developed a skin rash diagnosed as PE through skin pathology. Treatment involved oral methylprednisolone, intravenous human immune globulin, and supportive care, resulting in a cure within a month. This unique case highlights the importance of further research not only on lenvatinib but also on monitoring and managing adverse reactions associated with targeted drugs to optimize patient safety and treatment outcomes.

## 1 Introduction

Surgical resection or local ablation are essential components of curative treatment for hepatocellular carcinoma (HCC). Despite being considered ideal candidates for curative treatment, patients undergoing resection or ablation still face high rates of postoperative recurrence, with reported rates exceeding 70% within 5 years (1). Risk factors for early recurrence are primarily influenced by aggressive characteristics of the primary tumor, including tumor size, multiplicity, vascular invasion, high histological grade, and elevated serum α-fetoprotein levels (2). It is important to note that patients with microvascular invasion (MVI), defined by the presence of tumor cells within small blood vessels near the primary tumor, are at a greater risk of recurrence (3). Lenvatinib, an oral multikinase inhibitor, has shown significant clinical efficacy in the treatment of unresectable HCC and was approved by the United States Food and Drug Administration (FDA) as a first-line drug for this condition. The LANCE study (4), presented at the 2021 European Society for Medical Oncology (ESMO) annual meeting, revealed that the DFS in the group that received transarterial chemoembolization (TACE) alone was 9 months, whereas the group that received combined treatment achieved 17 months. However, lenvatinib has been associated with adverse effects such as hypertension, proteinuria, fatigue/asthenia, nausea, diarrhea, vomiting, stomatitis, and palmar-plantar erythrodysesthesia as reported in the REFLECT (5), Leap-002 (6) and BGB-A317-211 (7). Effective management of these adverse events is crucial during lenvatinib treatment. Recent studies have also highlighted the incidence and timing of common adverse events in lenvatinib-treated patients. For instance, Haddad et al. (8) reported that the most common adverse events associated with lenvatinib include hypertension, proteinuria, and fatigue, with most events being manageable with dose adjustments or supportive care. However, rare and severe adverse events, such as pemphigus erythematosus (PE), have not been extensively documented. This case report aims to contribute to the growing body of literature on lenvatinib-induced adverse events by presenting a unique case of PE in a patient with HCC.

## 2 Case report

A 68-year-old woman underwent a right total mastectomy and right axillary lymph node dissection at the University of Hong Kong Shenzhen Hospital on April 16, 2018. The postoperative pathological diagnosis revealed invasive ductal carcinoma of the right breast, grade 1, with a tumor diameter of approximately 1.8 cm. There was no clear invasion of blood vessels and nerves, with negative resection margins and negative sentinel lymph nodes. Immunohistochemistry results indicated positive ER (Allred score 8 points), positive PR (Allred score 8 points), negative HER-2, Ki-67 at 10%, and pathological stage pT1N0M0. Following surgery, she consistently took anastrozole 1 mg once daily until September 2022.

**Figure 1:**
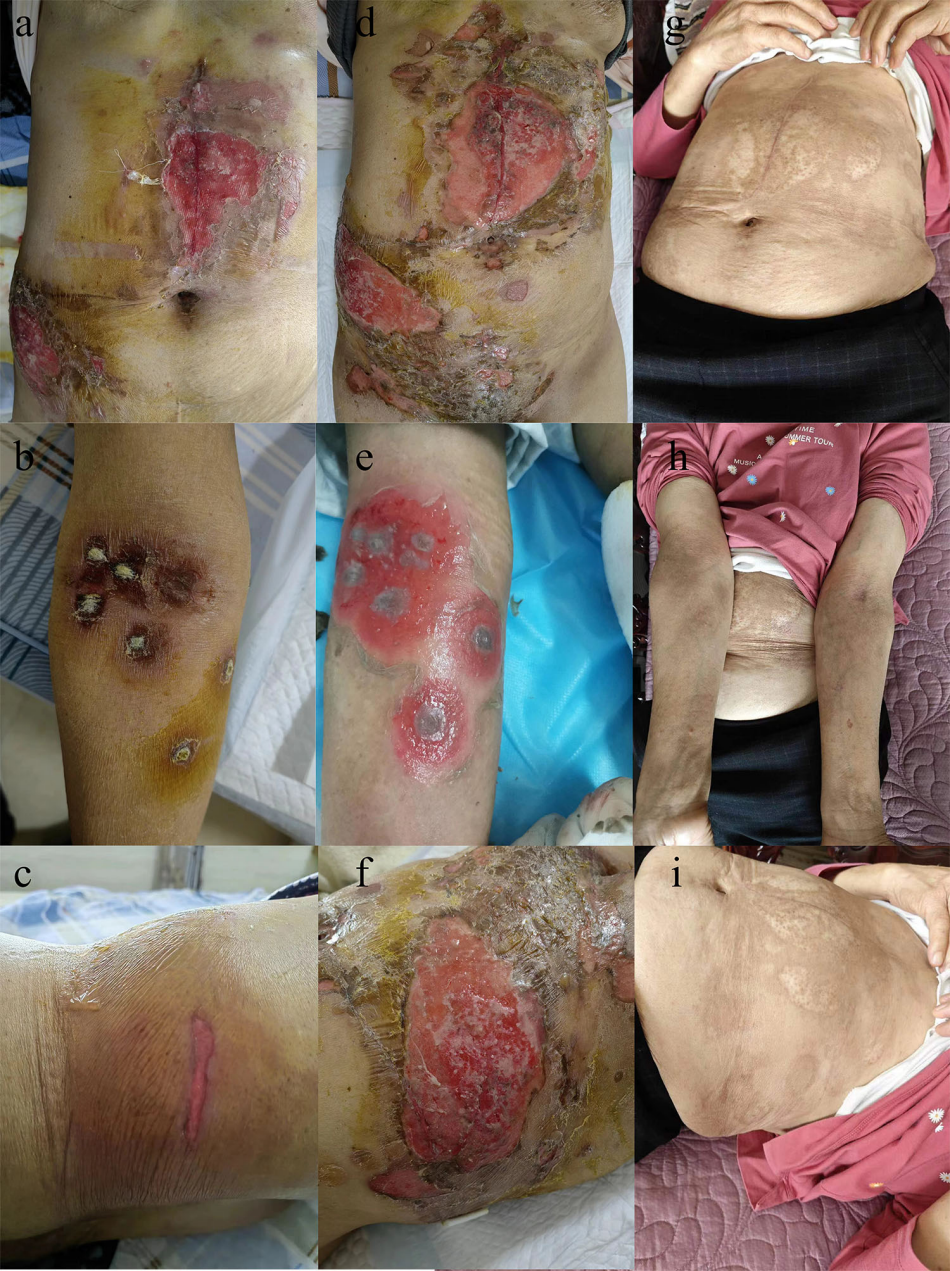
Following three weeks of oral administration of lenvatinib, the patient presented with a skin rash that initially manifested on the abdomen **(a)**, elbows **(b)** and waist **(c)**. The rash progressively spread to the trunk **(d)**, elbows **(e)**, waist **(f)**, perineum, limbs, and oral mucosa, accompanied by fluid exudation and numerous blisters. Over the treatment, the rash improved, blisters resolved, exudation decreased significantly, and trunk wounds healed **(g–i)**.

**Figure 2:**
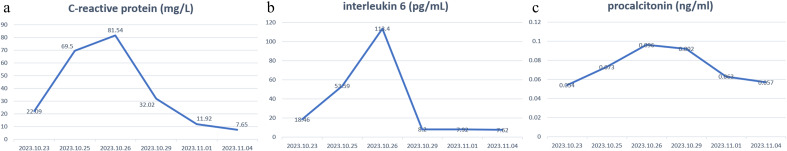
Changes of C-reactive protein levels **(a)**, interleukin 6 levels **(b)**, and procalcitonin levels **(c)** before and after treatment.

A PET-CT examination conducted on November 16, 2022, revealed a malignant tumor with a maximum diameter of around 4.1 cm, diagnosed with a metastatic tumor. Consequently, the patient commenced endocrine therapy with Abemaciclib in combination with Fulvestrant. Subsequent CT imaging on May 18, 2023, demonstrated tumor progression, leading to a switch to second-line treatment involving albumin-bound paclitaxel and capecitabine. Despite undergoing two rounds of chemotherapy treatment, the tumor continued to progress. Subsequently, the MRI revealed a tumor in the right lobe of the liver measuring approximately 13.9 cm×10.6 cm×17.2 cm. The presence of hepatic malignancy extending to the liver capsule was suspected, although the potential for other primary malignancies could not be ruled out. Following preoperative preparation and assessment, an anatomic right hemihepatectomy was performed on July 10, 2023, revealing HCC and a grade II Edmondson-Steiner classification at the National Cancer Center/Cancer Hospital & Shenzhen Hospital, Chinese Academy of Medical Sciences and Peking Union Medical College. Postoperative histopathology findings included a tumor diameter of 17 cm with serious microvascular invasion and tumor thrombus, but no satellite nodules were detected. Subsequent imaging tests showed no tumor recurrence, leading to TACE treatment on August 13, 2023. Prophylactic lipiodol-poppy plus pirarubicin hydrochloride was administered during the operation, with adjuvant oral Lenvatinib 8 mg daily initiated post-TACE. Following three weeks of oral administration of lenvatinib, the patient presented with a skin rash that initially manifested on the abdomen, elbows and waist (**Figures 1a–c**), after which it spread to other areas. The rash progressively spread to the trunk, perineum, limbs, and oral mucosa, accompanied by fluid exudation and numerous blisters (**Figures 1d–f**) quickly. Symptoms included pain, fever, and decreased blood pressure, with a peak body temperature of 38.7°C and blood pressure of 89/45 mmHg. After consulting dermatology and ruling out an allergic reaction, a skin biopsy was conducted on the left groin lesion. Microscopic examination revealed mild hyperplasia of the acanthous layer covered by squamous epithelium with hyperkeratosis and parakeratosis, as well as localized granular layer with increased basophilic granules and acanthosis. Intraepidermal blisters on the basal layer and acantholytic cells were observed. Inflammatory cells such as lymphocytes, histiocytes, plasma cells, and a small number of eosinophils and neutrophils were found infiltrating around dermal blood vessels. Immunofluorescence analysis indicated the presence of IgG (stratum spinosum +), IgM (-), IgA (-), C3 (stratum spinosum +), and Fib (-). The pathological assessment, which revealed changes consistent with pemphigus, suggested a diagnosis of pemphigus erythematosus (PE), aligning with the patient’s clinical presentation. Bacterial culture of the skin exudate revealed *Staphylococcus aureus* and multidrug-resistant *Escherichia coli* infection, with negative blood culture results. The patient’s routine blood tests, liver and kidney function, and thyroid function were mostly normal, except for hypoalbuminemia and hyponatremia. The C-reactive protein was 22.09 mg/L, anti-desmoglein 3 antibody was >150 U/ml, and total IgE antibody was 645.59 IU/mL. Fasting blood glucose was within normal limits, the fecal occult blood test was positive. Treatment included oral administration of 80 mg methylprednisolone, intravenous injection of human immune globulin and human albumin, amoxicillin and clavulanate potassium for infection, and topical application of mupirocin ointment and growth factors on the wound. C-reactive protein levels, interleukin 6 levels, and procalcitonin levels gradually normalized after 3 days of medication (**Figures 2a–c**). Over the following 2 weeks, the rash improved, blisters resolved, exudation decreased significantly, and trunk wounds healed (**Figures 1g–i**). Following complete resolution of the rash, the hormone dosage is gradually tapered down to maintain treatment until discontinuation of oral hormone therapy at the end of 6 months. Subsequent to the operation, intrahepatic tumor recurrence was detected through imaging during the 9-month follow-up, leading to the initiation of targeted therapy involving lenvatinib. However, a minor rash reappeared on the abdomen just 3 days after oral administration of lenvatinib, prompting immediate discontinuation of the treatment. Subsequent to the resolution of the rash, the patient commenced treatment with tremelimumab + durvalumab, which was well tolerated without any adverse reactions, including rash.

Based on the patient’s treatment history and timeline, this severe pemphigus was determined to be a grade IV adverse skin reaction according to the Common Terminology Criteria for Adverse Events 5.0 induced by lenvatinib.

## 3 Discussion

Lenvatinib, an oral multikinase inhibitor, has demonstrated significant clinical efficacy in the treatment of unresectable HCC. However, it is associated with a range of adverse effects, including hypertension, proteinuria, and skin reactions such as rash. Rash, as a common adverse event associated with the treatment of lenvatinib, has been mentioned in almost all clinical trials involving the drug, but the incidence was 10-12.5%, and almost all were < grade III severity (5-7). While most skin reactions are mild to moderate in severity, rare and severe reactions, such as pemphigus erythematosus (PE), have been reported. This case represents the first documented instance of PE induced by lenvatinib in a patient with HCC.

There has been a previous report of a 72-year-old man with lung metastasis of HCC received the first-line treatment with lenvatinib. Despite initial treatment with terbinafine tablets and luliconazole cream, the patient’s symptoms worsened, resulting in the development of a generalized erythematous rash (GER). Subsequent application of 10% urea cream led to the resolution of the rash within two weeks. However, upon reinitiating lenvatinib due to the progression of pulmonary metastases, the patient experienced erythema on the upper body along with itching. Discontinuation of lenvatinib and the use of difluprednate ointment and 10% urea cream resulted in improvement of the rash within two weeks (9). “Other reports of HCC patients who received lenvatinib and were diagnosed with pyoderma gangrenosum with lenvatinib-induced by histopathology demonstrated erosion and perivascular infiltration of neutrophils and lymphocytes in the upper dermis, cessation of lenvatinib led to rapid resolution after several weeks (10, 11).

Different from GER and pyoderma gangrenosum, pemphigus was a group of rare and severe autoimmune diseases clinically characterized by widespread erosion and blistering of the skin and mucous membranes (12). It was caused by pathogenic autoantibodies that attack two desmosomal adhesion proteins: desmoglein 3 and desmoglein 1. Depending on the clinical features, clinicians can categorize pemphigus into four subtypes: vulgaris, vegetans, foliaceus, and erythematosus. Pemphigus vulgaris (PV) was the most common and severe clinical variation (13). A benign variation of pemphigus foliaceus was known as PE. PE mainly involved photodistributed areas such as the face, scalp, back, and seborrheic areas of the upper trunk, which resemble cutaneous lupus erythematosus (12). Systemic glucocorticoids, with or without immunosuppressive medications, were used to treat severe PE. The most crucial element in treating pemphigus was systemic glucocorticoids with or without immunosuppressants (14). In this case, oral administration of lenvatinib was stopped immediately after the discovery of a large area of PE, and the scope of PE was not expanded after the administration of targeted drugs was stopped. Combined with the presence of dermatological history and no allergic history, the PE was thought to be responsible for the adverse drug reaction of lenvatinib.

The pathogenesis of lenvatinib-induced PE remains incompletely understood. It is plausible that lenvatinib or its metabolites act as haptens, binding to proteins and forming antigens that provoke immune responses, disrupting immune regulation, activating or impairing immune cells, and inciting autoimmune reactions against skin cells. Lenvatinib might modulate immune responses by influencing T cells, B cells, or natural killer cells, possibly activating autoreactive T cells that target self-antigens on skin cells, culminating in pemphigus-like lesions. Furthermore, lenvatinib could potentially disrupt intercellular junction proteins, like desmoglein, leading to compromised connections between skin cells and the development of pemphigus-like skin lesions. The mechanism might be attributed to the antiangiogenic effect of inhibiting vascular endothelial growth factor receptors (EGFR) and mast/stem cell growth factor receptor Kit, which promote apoptosis of keratinocytes by inhibiting the negative regulators of apoptosis and increasing the soluble Fas ligand concentration (15). By inhibiting the EGFR signaling pathway, lenvatinib affects the normal proliferation, migration, and differentiation of skin cells, destroys the skin barrier function, and may affect the immunomodulatory function of the skin, thus leading to the formation of skin inflammation and rash (16). Otherwise, the interaction between the patient’s history of breast cancer and prior endocrine therapy drugs with lenvatinib remains uncertain, raising questions about potential synergistic effects that could heighten the risk of adverse reactions, including PE.

This patient was diagnosed with hepatocellular carcinoma following a right hemihepatectomy, and the pathological examination revealed multiple high-risk recurrence factors, including microvascular invasion. Consequently, adjuvant treatment with lenvatinib was administered post-surgery in an effort to reduce the recurrence rate. However, the onset of severe pulmonary embolism interrupted the patient’s adjuvant therapy, leading to the discovery of intrahepatic recurrence 9 months after surgery, which was found to be in an advanced, unresectable state. After a thorough evaluation of the patient’s overall condition, tremelimumab in combination with durvalumab was chosen for treatment. Therefore, when selecting adjuvant therapy, it is imperative to consider the potential adverse reactions associated with drug treatment regimens and to implement effective management strategies.

To the best of our knowledge, this is the first documented case of PE caused by lenvatinib. Our approach to managing this situation involves: 1. Prompt discontinuation of the tyrosine kinase inhibitor drug; 2. Immediate skin biopsy for lesion type determination; 3. Culturing local exudate for bacterial susceptibility testing to guide antibiotic therapy selection; 4. Implementing local and systemic supportive treatments to aid in recovery. This study serves as a reminder to healthcare professionals about the importance of closely monitoring patients for abnormal manifestations following the administration of lenvatinib for HCC. While this case may present unique characteristics in comparison to other drug-induced adverse skin reactions, it underscores the diverse and complex nature of adverse drug reactions.

This case highlights the importance of monitoring patients for rare but severe adverse reactions during lenvatinib treatment. Further research is needed to elucidate the mechanisms underlying lenvatinib-induced PE and to develop strategies for preventing and managing such adverse events.

## Data Availability

The original contributions presented in the study are included in the article/supplementary material. Further inquiries can be directed to the corresponding authors.
